# Use of Immune Checkpoint Inhibitors in Elderly Patients With Chronic Kidney Disease and Renal Cell Carcinoma Metastasis of the Parotid Gland: Case Report and Review of the Literature

**DOI:** 10.7759/cureus.28868

**Published:** 2022-09-06

**Authors:** Andreea Parosanu, Cristina Orlov Slavu, Cristina Pirlog, Ioana M Stanciu, Cornelia Nitipir

**Affiliations:** 1 Oncology, Elias Emergency University Hospital, Bucharest, ROU

**Keywords:** immune checkpoint inhibitors, therapeutic strategies, immunohistochemistry, parotid gland metastasis, clear renal cell carcinoma

## Abstract

Renal cell carcinoma metastasis of the parotid gland is extremely rare, and the present literature review found only 23 cases reported in the last two decades. We present a case of a 75-year-old woman with a colon cancer history who had parotid gland metastasis as the first manifestation of second primary kidney cancer. The presence of multiple comorbidities affected medical decision-making. Therefore the patient was treated primarily with immunotherapy. After four cycles of dual immune checkpoint blockade, this advanced patient still benefited and was changed to nivolumab monotherapy as maintenance therapy.

## Introduction

Over 14,000 patients are diagnosed with stage IV kidney cancer annually [1]. Renal cell carcinoma initially metastasizes to the lungs, liver, and bones [2]. However, the parotid glands may also be affected by metastatic kidney cancer, accounting for approximately 0.5% of the cases [3]. Metastases in the parotid glands have been observed in patients with widespread metastatic disease. Nonetheless, extremely few cases of solitary involvement have been reported in the literature to date [4]. We discuss the challenges associated with the diagnosis and treatment of a patient with parotid metastasis as the initial sign of renal cell carcinoma.

## Case presentation

A 75-year-old woman with multiple comorbidities, including hypertension, coronary artery disease, type 2 diabetes, and stage IV diabetic nephropathy, reported a painful left parotid mass to the department of otorhinolaryngology. Although the patient had undergone a curative-intent surgery for stage II right colon carcinoma in 2016, she neither received postoperative treatment nor a follow-up. Furthermore, blood screening revealed mild anemia (10.8 g/dL; normal range =12-15 g/dL) and stage 3B of chronic kidney disease, with a decreased estimated glomerular filtration rate (eGFR) up to 40 mL/min.

**Figure 1:**
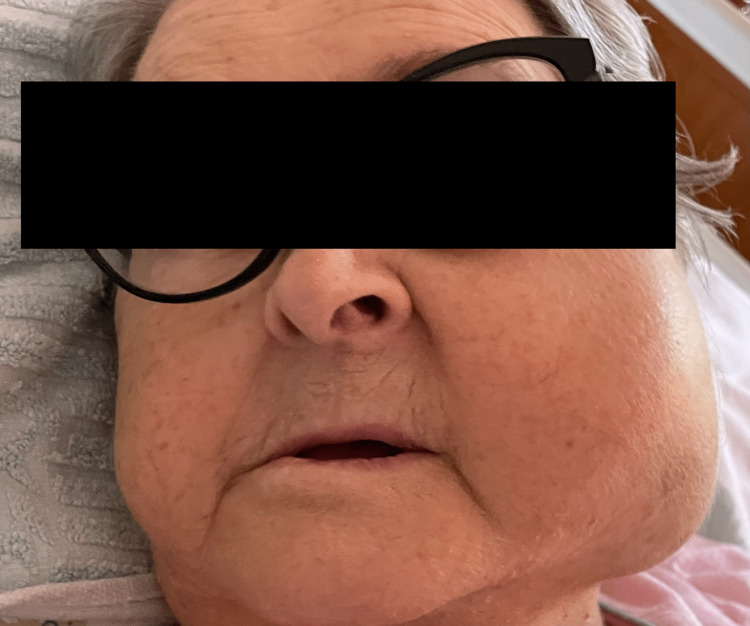
The patient at initial examination, showing a large tumor of the left parotid gland

Clinical examination indicated that the patient was hemodynamically and respiratory stable, with no weight loss but with a painful mass within the left jawbone accompanied by left hemifacial weakness (Figure 1). In addition, the patient revealed the tumor had been present for more than seven months before she sought medical attention.

**Figure 2:**
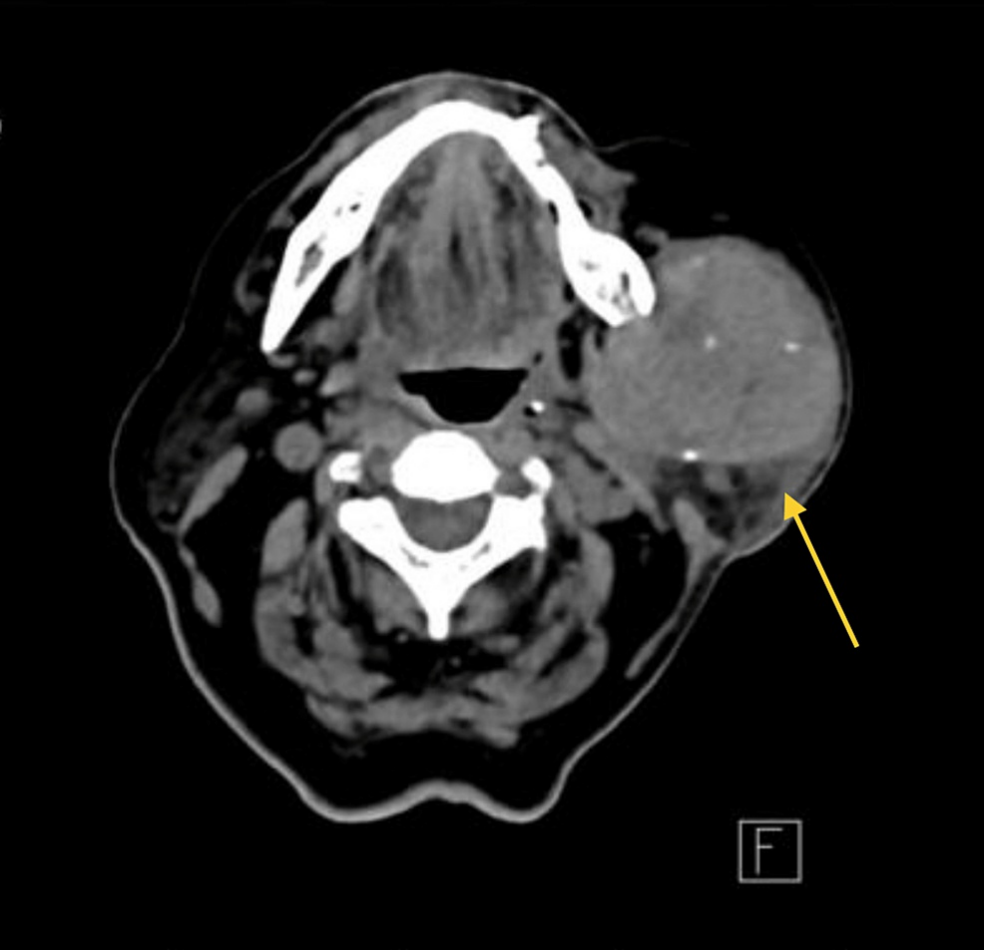
A well-defined tumor with calcification was identified in the left parotid gland

Moreover, ultrasonographic examination and computed tomography (CT) scan suggested a slightly hypoechogenic benign tumor, with well-defined, smooth margins and moderate vascularization, confined to the left parotid lodge (Figure 2).

**Figure 3:**
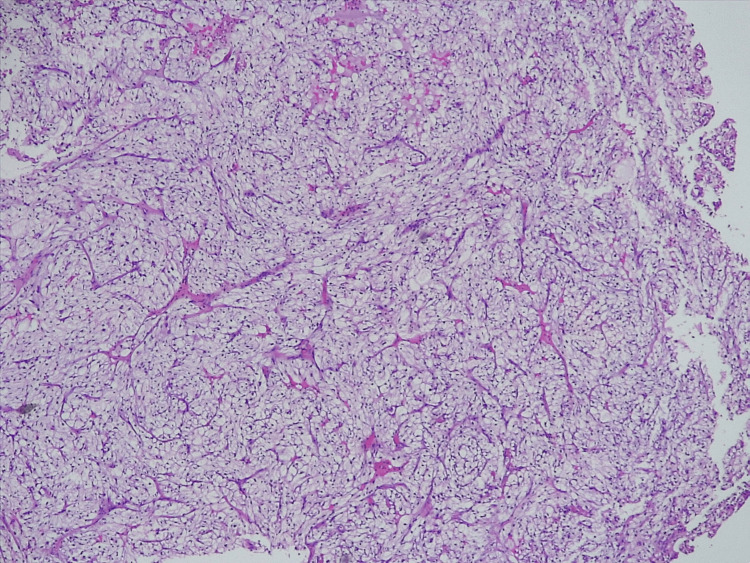
Hematoxylin and eosin stain of metastatic clear cell renal cell carcinoma

The patient underwent a left parotid biopsy, and the samples were subjected to histopathological and immunohistochemical analyses. Conventional hematoxylin and eosin staining revealed carcinoma with clear-cell histomorphology, whereas immunohistochemistry indicated that tumor cells were positive for CD10 and carbonic anhydrase IX (CA-IX) and negative for cytokeratin 7 (CK7) (Figure 3). Therefore, the patient was diagnosed with parotid metastasis from renal cell carcinoma.

**Figure 4:**
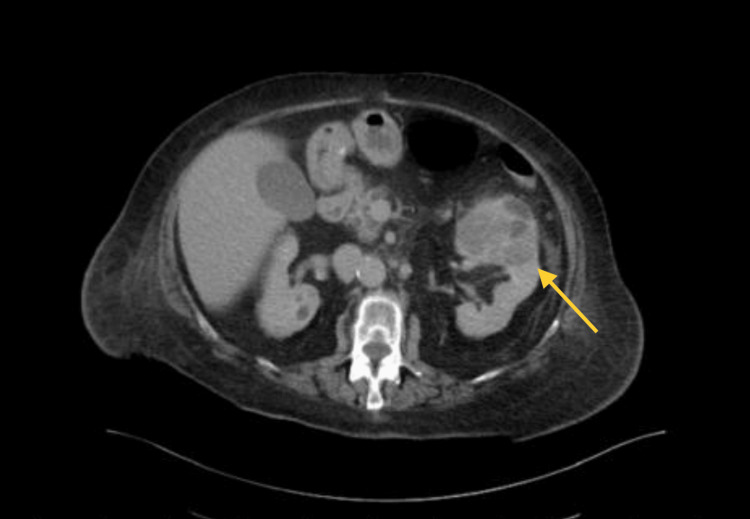
A computed tomography scan showed a left kidney mass (arrow) with features suggestive of primary renal cell carcinoma

Because positron emission tomography (PET) imaging was not eligible for reimbursement in our country, we decided to perform a whole-body CT scan. The results confirmed the presence of a 7×5 cm tumor in the left kidney (Figure 4). We also observed a few osteolytic metastases in the thoracic vertebrae and simple bilateral kidney cysts. In addition, bone scintigraphy verified the metastatic lesions in the lower thoracic and upper lumbar vertebrae.

Given the clinical, biological, and histological findings, intermediate-risk metastatic renal cell carcinoma was diagnosed. The patient exhibited only one International Metastatic Renal Cell Carcinoma Database Consortium (IMDC) risk factor - a mild, normocytic, normochromic anemia (Hb =10.8 g/dL; normal range =12-15 g/dL). The Multidisciplinary Tumor Board recommended the initiation of double immunotherapy (nivolumab 3mg/kg and ipilimumab 1mg/kg administered every three weeks for four doses), zoledronic acid with dose adjusted for creatinine clearance, and palliative radiation therapy (300 cGy in five fractions) for the parotid tumor. After 12 weeks of therapy, the cervical-thoracic-abdominal-pelvic contrast-enhanced CT scan demonstrated favorable outcomes, decreasing the size of parotid and kidney tumors by at least 15%. Moreover, the patient had stable disease according to bone scintigraphy. Presently, the patient is undergoing systemic monotherapy with nivolumab (240 mg administered every two weeks) with no adverse reactions.

## Discussion

To the best of our knowledge, the parotid gland is not a common primary site for metastatic disease. Approximately 5% of salivary gland metastases result from renal cell carcinoma, making an accurate diagnosis in such cases essential [5].

We also performed a literature survey using the PubMed database to retrieve case reports on the occurrence of parotid metastases from renal cell carcinoma and selected those reported in English between January 2000 and August 2022 (Table 1). 

**Table 1:** Case reports on the occurrence of parotid metastases from renal cell carcinoma

References	Age	Sex	Other metastases	Time from diagnosis to parotid metastasis (months)	Case particularities
Spreafico et al., 2008 [5]	69	F	None	24	
Franzen et al., 2018 [6]	74	F	Controlateral parotid gland	72	History of colorectal cancer stage III
Kolokythas et al., 2015 [7]	83	M	None	120	
Balaban et al., 2009 [8]	66	F	Liver, lung, brain	18	
Udager et al., 2014 [9]	64	M	Lung	72	
Yanlan et al., 2013 [10]	44	F	Bone, lung	0	Parotid metastasis as initial presentation
Halbony et al., 2022 [11]	50	M	Bone	0	Parotid metastasis as initial presentation
Singla et al., 2022 [12]	59	M	None	48	
Lieder et al., 2017 [13]	74	-	None	78	
Majewska et al., 2016 [14]	66	F		0	Parotid metastasis as initial presentation
	76	F	None	0	Parotid metastasis as initial presentation
	68	M	None	0	Parotid metastasis as initial presentation
	69	M	None		Parotid metastasis as initial presentation
	-	M	Lung	-	
	-	F	Bilateral kidney	11	
	60	F	None	156	
Maralani et al., 2014 [15]	64	F	Bilateral parotid, submandibular and thyroid glands	0	Parotid metastasis as initial presentation
Deeb et al., 2012 [16]	82	F	None	228	History of multifocal renal cell carcinoma and chronic lymphocytic leukemia
Miah et al., 2010 [17]	61	F	Sbmandibular and thyroid glands	84	History of breast cancer
Mrena et al., 2008 [18]	58	F	None	0	Parotid metastasis as initial presentation
	76	F	Lung, bone	108	
	62	M	None	60	
Hussain et al., 2016 [19]	65	F	None	96	Parotid metastasis as initial presentation

This report presents the case of an elderly female patient with atypical parotid metastasis as the initial sign of renal cell cancer. We revealed that only nine similar cases have been reported previously. Furthermore, according to the survey, parotid metastasis was detected within seven years of the initial diagnosis. However, Deeb et al. (2012) reported a rare parotid metastasis more than 19 years after initial diagnosis [16]. In addition, the average age at the time of diagnosis of parotid metastasis was approximately 66 (44-82) years, with a higher incidence in women compared to men (1.75:1).

Although primary parotid cancer and parotid metastases from renal cell carcinoma exhibit similar clinical signs, such as abnormal growing mass in the preauricular region and occasional compression symptoms, their management varies considerably. Consequently, a precise diagnosis is crucial. Moreover, our patient exhibited no signs to suggest renal cell carcinoma, and the CT scan findings were the primary indicators of kidney cancer.

This case was also an example of second primary kidney cancer. The patient in this study developed renal cell carcinoma eight years after a well-differentiated stage II colon adenocarcinoma. Previous reports indicate patients with second primary kidney cancer after breast cancer, colon cancer, and leukemia [6, 16,17].

Even though imaging plays an essential role in the diagnosis of primary tumors and other metastases, the pathological examination is the most important tool for diagnosis and decision-making. The histological findings of the parotid tumor biopsy indicated clear-cell carcinoma. Nonetheless, colon carcinoma metastasis could not be excluded because of the oncological history of the patient. Therefore, the differential diagnosis must be established with other clear-cell tumors, including clear-cell salivary gland tumors and carcinomas from the thyroid gland, colon, prostate gland, kidneys, and liver [20]. In this regard, immunohistochemical studies play an important role. CD10 is the most sensitive and diagnostically helpful marker for clear-cell renal carcinoma. Other distinguishing features include the overexpression of carbonic anhydrase IX (CA-IX) and the downregulation of cytokeratin 7 (CK7) [21].

Diagnosis of metastatic renal cell carcinoma influences the therapeutic management of palliative care and requires a multidisciplinary approach. Several therapeutic alternatives exist to treat metastatic renal cell carcinoma of intermediate risk, including tyrosine-kinase inhibitor (TKI) monotherapies and immunotherapy-based combination treatments [22]. Unfortunately, the preexisting severe cardiac disease altered the patient's ability to undergo nephrectomy or parotid metastasectomy, and the multidisciplinary team recommended systemic therapy. Even if systemic therapy of metastatic kidney cancer changed annually with newer drug combinations supplanting older regimes, we had to consider therapeutic options available in our country. Unfortunately, at that time, we did not have access to novel TKI/immune checkpoint inhibitor combinations, and the patient started treatment with nivolumab plus ipilimumab.

Furthermore, this case was challenging because the patient was diagnosed with chronic kidney disease. While patients with chronic kidney disease or those on dialysis were excluded from phase III clinical trials, several studies confirmed the safety profile of immune checkpoint inhibitors (ICI) in this particular population [23]. For example, a recent review that included cancer patients with end-stage kidney disease receiving immune checkpoint inhibitors found that over 80% of the patients achieved partial or complete responses with minimal immune-related adverse events [24]. Ansari et al. reported one patient on dialysis with metastatic RCC who experienced over 22 months of progression-free survival from immunotherapy with nivolumab [25]. Similarly, Ito and his colleagues confirmed a complete clinical response to nivolumab in an elderly patient with metastatic renal cell carcinoma and end-stage renal disease [26]. Moreover, proteolytic enzymes metabolize nivolumab and ipilimumab into polypeptides and amino acids. As a result, renal impairment does not affect their pharmacokinetics, and the dose of these drugs need not be adjusted [27]. This data suggested that immune checkpoint inhibitors were the best first-line treatment for the patient in this study. Therefore, the patient started double immunotherapy with nivolumab plus ipilimumab, zoledronic acid for bone metastases, and external radiotherapy for left parotid gland metastasis. Follow-up imaging scans after four cycles of double immunotherapy showed a favorable response with a significant decrease in renal and parotid tumors and stable metastatic bone disease. Currently, the patient is continuing maintenance monotherapy with nivolumab without any toxic side effects. Nevertheless, in the face of limited resources in our hospital, we will measure the response to treatment after another four cycles of immunotherapy using CT scans and Response Evaluation Criteria in Solid Tumors (RECIST) criteria. Furthermore, we will closely monitor the renal function and for underlying immunotherapy-related adverse events. 

## Conclusions

As only a few cases of parotid metastasis from renal cell carcinoma have been described, this case contributes to the limited data reported in the literature. This report also highlights the impact of comorbidities on cancer treatment. Despite advancements in metastatic renal cell carcinoma, one significant obstacle might be the accessibility to the newest treatments in our country. However, analyzing the risks and benefits of different treatment options helped us decide on the best treatment plan.
